# Endothelial Glycocalyx-Mediated Intercellular Interactions: Mechanisms and Implications for Atherosclerosis and Cancer Metastasis

**DOI:** 10.1007/s13239-020-00487-7

**Published:** 2020-09-30

**Authors:** Solomon A. Mensah, Alina A. Nersesyan, Eno E. Ebong

**Affiliations:** 1 Department of Bioengineering, Northeastern University, Boston, MA USA; 2 Department of Chemical Engineering, Northeastern University, 360 Huntington Avenue, 335 Interdisciplinary Science and Engineering Complex, Boston, MA 02115 USA; 3 Department of Neuroscience, Albert Einstein College of Medicine, New York, NY USA; 4 Present Address: Department of Biomedical Engineering, Worcester Polytechnic Institute, Worcester, MA USA

**Keywords:** Glycocalyx, Endothelial cells, Heparan sulfate, Sialic acid, Cancer metastasis, Atherosclerosis

## Abstract

**Purpose:**

The endothelial glycocalyx (GCX) plays a critical role in the health of the vascular system. Degradation of the GCX has been implicated in the onset of diseases like atherosclerosis and cancer because it disrupts endothelial cell (EC) function that is meant to protect from atherosclerosis and cancer. Examples of such EC function include interendothelial cell communication via gap junctions and receptor-mediated interactions between endothelial and tumor cells. This review focuses on GCX-dependent regulation of these intercellular interactions in healthy and diseased states. The ultimate goal is to build new knowledge that can be applied to developing GCX regeneration strategies that can control intercellular interaction in order to combat the progression of diseases such as atherosclerosis and cancer.

**Methods:**

*In vitro* and *in vivo* studies were conducted to determine the baseline expression of GCX in physiologically relevant conditions. Chemical and mechanical GCX degradation approaches were employed to degrade the GCX. The impact of intact versus degraded GCX on intercellular interactions was assessed using cytochemistry, histochemistry, a Lucifer yellow dye transfer assay, and confocal, intravital, and scanning electron microscopy techniques.

**Results:**

Relevant to atherosclerosis, we found that GCX stability determines the expression and functionality of Cx43 in gap junction-mediated EC-to-EC communication. Relevant to cancer metastasis, we found that destabilizing the GCX through either disturbed flow-induced or enzyme induced GCX degradation results in increased E-selectin receptor-mediated EC-tumor cell interactions.

**Conclusion:**

Our findings lay a foundation for future endothelial GCX-targeted therapy, to control intercellular interactions and limit the progression of atherosclerosis and cancer.

## Personal Reflections to Honor Dr. John Tarbell

### Reflection from Eno E. Ebong

First, I thank the Cardiovascular Engineering and Technology (CVET) journal guest editors Keefe Manning and Hanjoong Jo, for assembling this special issue to celebrate the lifetime achievements of John Tarbell. I am honored to have been selected to contribute to this issue with an article that summarizes the dissertation of one of my first PhD students, Solomon Mensah, who I initially met in Dr. Tarbell’s Laboratory.

I first met John Tarbell in 2006 after I had received my PhD degree in Biomedical Engineering from the Rensselaer Polytechnic Institute. I had completed this degree following studies in Mechanical Engineering as an undergraduate student. Due to my strictly Mechanical Engineering background, I had to climb a steep learning curve during my PhD studies. Under Natacha DePaola’s supervision, I successfully climbed the learning curve on a project involving endothelial cell biology and physiology, mechanotransduction, and gap junctional communication.^28^ Upon completion of my dissertation and in considering postdoctoral opportunities, I decided that I was interested in transitioning to an experience that would teach me more about cell biology and other aspects of life science. I was specifically interested in learning molecular biology, pathology, animal modeling, and other approaches that could be applied to studying cardiovascular mechanobiology and atherosclerosis. At a Biomedical Engineering Society (BMES) meeting, I crossed paths with Sheldon Weinbaum at a poster presentation of my dissertation project. He pointed out that my project had completely ignored the glycocalyx (GCX), and upon learning about my postdoctoral research interests, he directed me to apply for a position in the laboratory of John Tarbell.

I was pleased when Dr. Tarbell invited me to work with him as a postdoctoral fellow. Dr. Tarbell proposed a collaborative, interdisciplinary, and multi-institutional postdoctoral experience. I was thrilled when he proposed that he would co-advise me in the College of Engineering at the City College of New York while Dr. David Spray would co-advise me at the Albert Einstein College of Medicine in New York, as Drs. Tarbell and Spray are both pioneers and world renown in their respective fields. John added that I would receive funding and state-of-the-art training provided by Albert Einstein’s Mechanisms of Cardiovascular Disease Training Grant (NIH T32). This was an opportunity that I could not pass on, and I enthusiastically joined his team in 2007.

**Figure 1:**
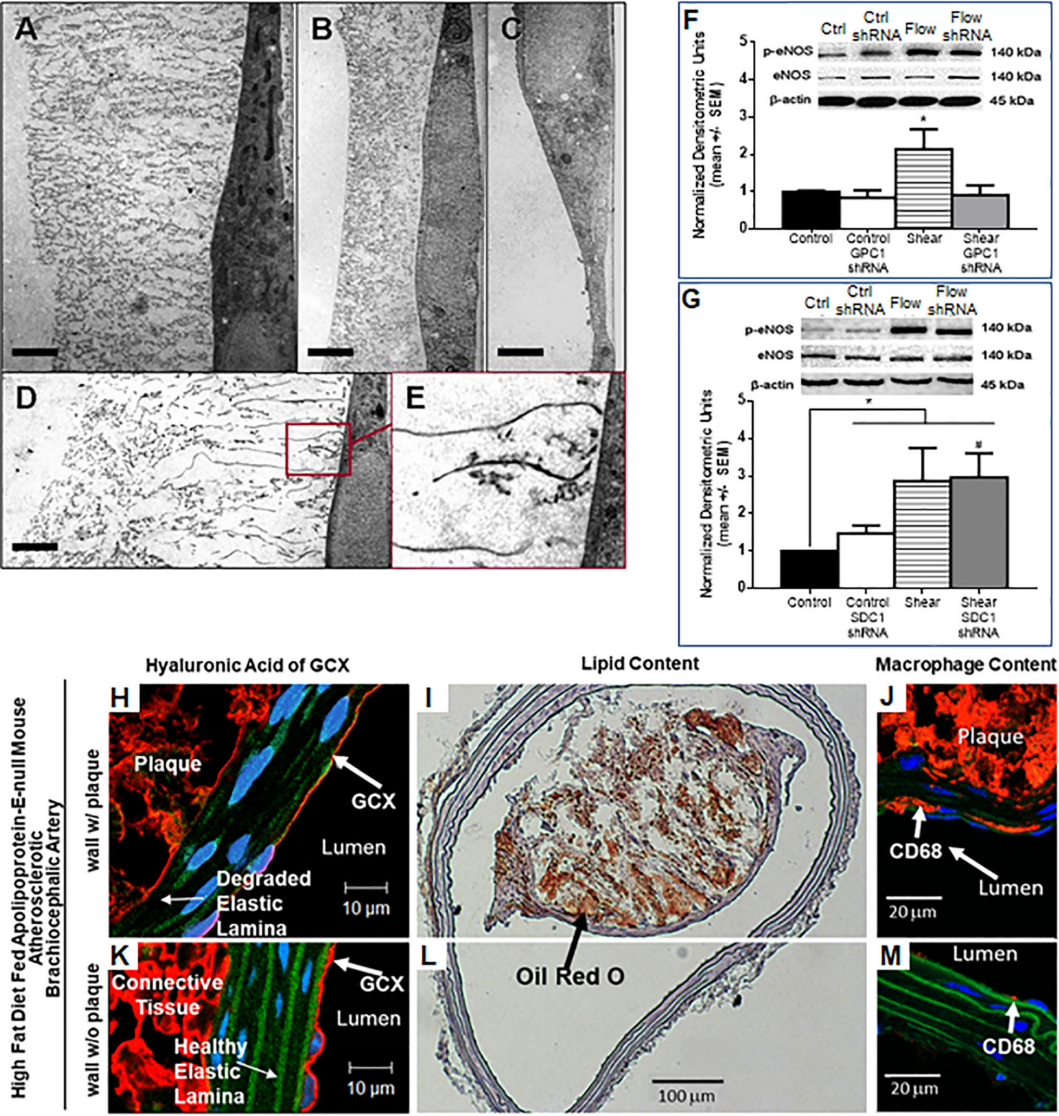
Reflection on the work that we performed while in John Tarbell’s laboratory. (**a–e)** We were the first to use rapid freezing/freeze substitution transmission electron microscopy for optimal preservation the endothelial GCX *in vitro*. (**a)** GCX in no flow conditions on bovine aortic ECs. Bar = 2 µm. (**b)** GCX in no flow conditions on rat fat pad ECs. Bar = 2 µm. (**c)** Bovine aortic EC GCX is lost in no flow when protein is depleted from the culture media. Bar = 2 µm. (**d, e)** In flow conditions bovine aortic EC GCX exhibits alignment of elements in a 2-3 µm region close to the cell membrane. Bar = 2 µm. (**f–g)** We identified that glypican **(f)**, and not syndecan **(g)**, is the heparan sulfate-bound core protein that is responsible for mediating bovine aortic EC 3-hour flow-induced expression of activated (phosphorylated) endothelial nitric oxide synthase (p-eNOS). In the 3-hour flow period, total eNOS did not change (β-actin was probed as the loading control). (**h–m)** We used an atherosclerosis animal model to show that GCX **(h and k)** shedding initiated by inflammation facilitates vessel wall infiltration and retention of lipids **(I and l)** and monocyte-derived macrophages **(j and m)** that contribute to the development of atherosclerotic plaques. *Data presented in this figure are reused with permission from previous publications:.*^14^,^29^,^30^

We studied the structure of the endothelial surface GCX and its role in endothelial cell remodeling and mechanobiology in response to fluid shear stress. In one study, we applied rapid freezing/freeze substitution transmission electron microscopy as a novel approach to defining the ultrastructure of the endothelial surface GCX and its changes as a result of the macro- or micro-vessel origin and due to the bio-chemical and -mechanical environment (Figs. 1a to 1e).^30^ In another study, RNA interference techniques, fluorescent biomarkers, confocal microscopy, and protein biochemistry were applied to identify the GCX core proteins that are responsible for the mechanobiology of shear-induced nitric oxide (Figs. 1f and 1g), a fundamental vascular control mechanism of great importance in health and disease, and endothelial cell remodeling in response to shear stress (alignment and elongation in the direction of shear).^29^ In a third study, which served as a platform for me to mentor one of John’s many undergraduate research assistants, Solomon Mensah (see his reflection below), we used a pre-clinical animal model to show that GCX shedding initiated by inflammation facilitates vessel wall infiltration and retention of lipids and other components that contribute to the development of atherosclerotic plaques (Figs. 1h to 1m).^14^ I could go on about other studies, but instead I will end here and summarize by saying that my experience as John Tarbell’s postdoctoral trainee was invaluable and laid a strong foundation for me to develop my own independent research program as a faculty member at Northeastern University. My research program excels at operating at the interface of engineering and life science, is highly collaborative involving experts from multiple disciplines and institutions, and promises to achieve clinical translation and improved human cardiovascular health.

**Figure 2:**
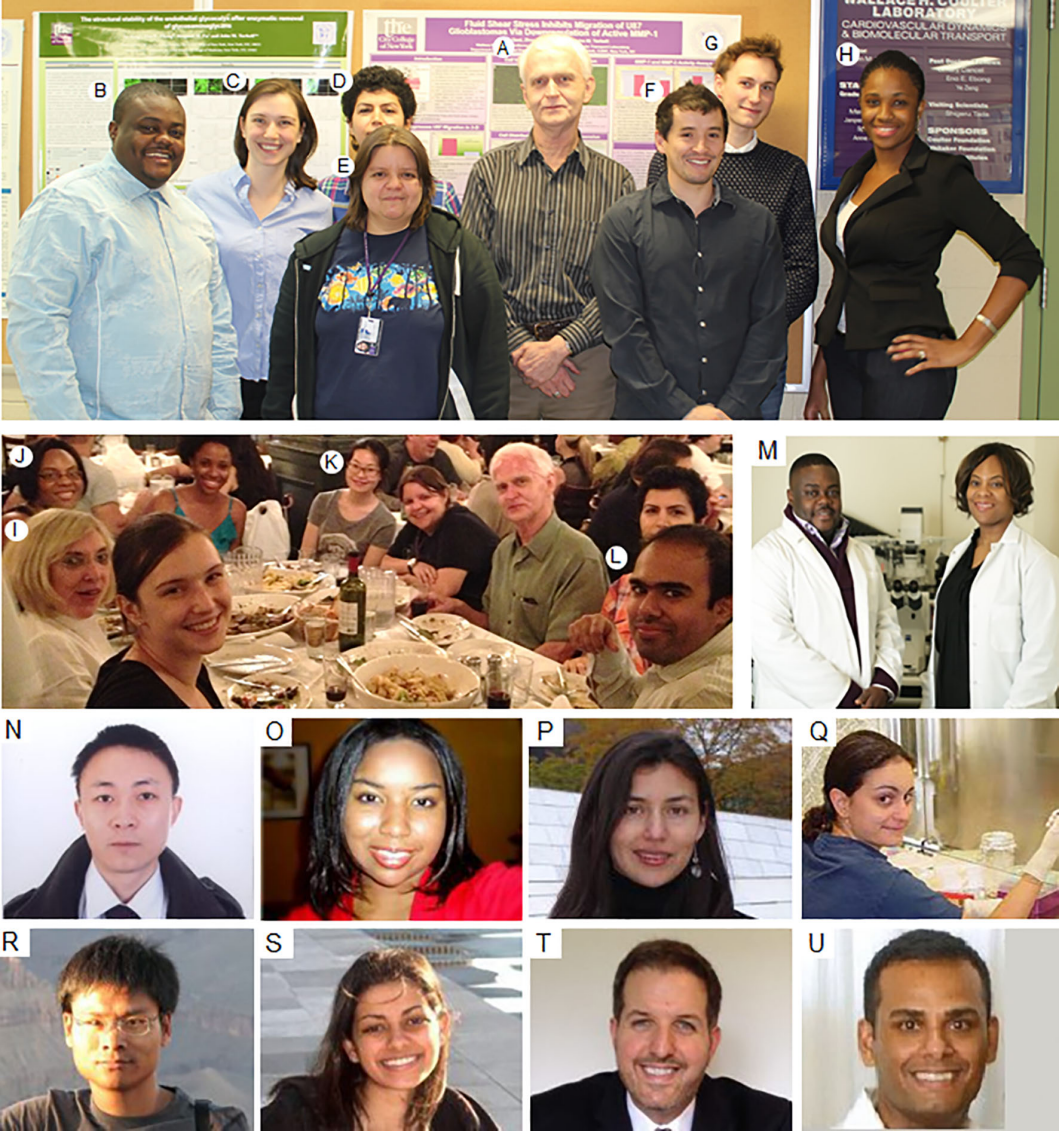
While working in John Tarbell’s lab, we experienced a diverse and inclusive research group. This figure presents Tarbell research family members who we overlapped with. (**a)** John Tarbell. (**b)** Solomon Mensah. (**c)** Anne Marie W. Bartosch. (**d)** Maria Nikmanesh. (**e)** Limary Cancel. (**f)** Ronny Amaya. (**g)** Louis Hennequin. (**h)** Sparkle Russell-Puleri. (**i)** Kathy Tarbell. (**j)** Eno Essien Ebong. (**k)** Hongyan Kang. (**l)** Henry Qazi. (**m)** Solomon Mensah (on left) and Eno Ebong (on right) immediately after moving on from John Tarbell’s lab and starting up a new lab at Northeastern University. (**n)** Ye Zeng. (**o**) Michele Waters. (**p**) Sandra Veronica Lopez-Quintero. (**q**) Danielle E. Berardi. (**r**) Zhong-Dong Shi. (**s**) Giya Abraham. (**t**) Jeff S. Garanich. (**u**) Rishi Mathura.

We are writing this manuscript against the backdrop of global civil protests against systemic racism, implicit and unconscious bias, and micro- and macro-aggressions that plague our dear United States and other countries. Therefore, I would be remiss if I did not reflect on John Tarbell’s contributions to diversity, equity, and inclusion, namely by pointing out that during my tenure in John Tarbell’s laboratory he recruited and supported an extremely diverse cadre of researchers, including underrepresented minorities like myself as his postdoctoral trainee and Solomon Mensah as his undergraduate research assistant (Fig. 2). We were not just props or filling a quota in John Tarbell’s laboratory, which is the experience of many underrepresented minorities who are engaged in science, technology, engineering, and mathematics (STEM) undergraduate, doctoral, and postdoctoral training programs. Instead, we were welcomed, encouraged, and engaged, and our intellectual input was valued. For example, I personally felt and continue to feel valued any time when Dr. Tarbell asks me to give a talk on his behalf or invites me to a scientific meeting to meet with pioneers and leaders in our field. For this, I owe many thanks to John Tarbell.

In honor of John Tarbell, herein, I and Solomon Mensah, along with our research team member Alina Nersesyan, provide a brief review of research that took place shortly after we moved on from John Tarbell’s laboratory. The research project elucidates the GCX-mediated mechanobiology role in regulating intercellular interactions at the blood vessel wall to prevent or promote conditions relevant to atherosclerotic cardiovascular disease and cancer.

Other medical conditions, such as diabetes, inflammation and vasculitis, sepsis, and ischemia/reperfusion,^57^,^73^,^75^,^87^ are known to depend on or affect GCX-mediated mechanobiology, in many cases in conjunction with intercellular interactions. Although discussion of these medical conditions is important, it is outside of the scope of this review paper and addressed elsewhere within this special issue and in other publications.^57^,^73^,^75^,^87^

### Reflection from Solomon A. Mensah

The first time I met John Tarbell was in 2009 in the hallways of the Biomedical Engineering Department at the City College of New York. I had just immigrated from Ghana in West Africa and I was hoping to get admitted into the College of Engineering. At that time, I wasn’t sure of my major and I remember vividly asking him what the role of biomedical engineers were in American society. He graciously explained in detail the essence of biomedical research and discussed his research on endothelial GCX, which got me very interested.

After my admission, I joined his laboratory and conducted my undergraduate research with his team until I graduated. I was very fortunate to have been awarded a prestigious National Institutes of Health (NIH) undergraduate research fellowship under John Tarbell’s supervision. I was also fortunate that Dr. Tarbell introduced me to his postdoctoral trainee, Eno Essien Ebong, who was assigned as one of my mentors in the lab. The research foundation and guidance that I initially received from working with Dr. Tarbell and his students led to my PhD studies. Since then, I have seen myself complete my PhD studies and bud into a young independent investigator, and I am currently a Future Faculty Postdoctoral Fellow and Adjunct Professor at Worcester Polytechnic Institute.

I owe my research career to John Tarbell and Eno Ebong and I am grateful for this opportunity to publish this paper in Dr. Tarbell’s honor.

## Introduction: Why Study Glycocalyx-Mediated Intercellular Interactions?

Atherosclerotic cardiovascular disease and cancer are the two leading causes of death^117^ and are responsible for 50% of deaths worldwide annually.^11^ Atherosclerosis and cancer metastasis are both characterized by modifications in the host blood vessels, due to inflammation and change in intercellular interactions. Atherosclerosis is distinguished by plaque formation as a result of accumulation of cholesterol and cellular debris within the vascular wall, which leads to vessel damage.^13^ On the other hand, cancer metastasis is distinguished by the formation of secondary tumors away from the location of the primary tumor. The process of secondary tumor formation involves circulating tumor cell (CTC) escape from the primary tumor and survival in the bulk flow before extravasation and re-growth occurs in the microenvironment of the secondary organ.^64^ Recent reports by Suzuki *et al.* and others suggest that patients with atherosclerotic plaques could be at a higher risk for developing cancer.^19^,^32^,^103^ Other reports indicate that long-term cardiovascular risk attenuates cancer survival rate and efficacy of early cancer therapy, particularly with respect to breast cancer, which has been the cancer of focus in our research laboratory.^36^,^53^ Furthermore, it is known that cardiovascular disease contributes to a majority of the deaths amongst breast cancer patients.^15^ Cardiovascular disease and cancer demonstrate similar pathophysiological symptoms such as inflammation as mentioned above,^6^,^80^ neovascularization,^33^,^74^ and epigenetics in the form of DNA methylation and chromatin remodeling.^94^,^118^ These two diseases also share some common risk factors which include obesity and hypertension.^32^ The commonalities and connections between atherosclerosis and cancer suggest that they could develop via common cellular and molecular pathways.^32^

One common cellular and molecular pathway is thought to involve endothelial cells (ECs) and their GCX. The GCX is a hydrated sugar-rich layer coating the ECs, making it a lining for the inside of blood vessels.^123^ GCX dysfunction results in the lack of proper control of intercellular interaction between individual ECs with adjacent ECs and with circulating cells, including inflammatory cells and CTCs, leading to disease progression.^14^,^70^ The modes of intercellular interactions are many. However, those mediated by connexin (Cx) proteins that form gap junction channels and adhesion molecules that form receptor-ligand bonds will be the focus of this review paper. These structures are of particular interest based on their physical proximity to the GCX and their implications in the onset and progression of atherosclerosis and metastatic cancer. Furthermore, the role of the GCX in mediating EC interactions with its adjacent cells and with CTCs has not been fully clarified. It is important that the role of the GCX in regulating intercellular interactions be clarified to strengthen our understanding of the GCX-mediated mechanisms that contribute to either healthy or disease conditions, and to eventually lead to the development of novel GCX-targeted drugs to address GCX degradation and treat atherosclerosis and cancer.

## Intercellular Interactions in Normal Physiology, Atherosclerosis, and Cancer

### Overview

Generally, endothelium interactions with neighboring cells are mediated by different routes which include contact with other cells via integrins,^59^,^79^ junctional proteins,^10^,^120^ adhesion molecules,^4^,^65^ extracellular vesicles^47^,^114^ or the secretion of proteins^44^ and cytokines^38^ into the extracellular space. Integrins are transmembrane proteins that function as receptors for extracellular ligands, and play significant roles in vascular development and vascular health.^89^ The clustering of integrins results in the formation of focal adhesion complexes which form mechanical connections between intracellular cytoskeleton and extracellular substrates.^90^ In addition, integrins function as signal transduction molecules that can control intracellular pathways to regulate cellular activities. Junctional proteins mediate the adhesion and interaction between adjacent ECs, and these junctional proteins include tight junctions, adherence junctions and gap junctions.^9^ The expression of these junctional proteins depend on the tissue type and the communication requirement between the cells.^9^ ECs also interact with themselves and others, i.e. CTCs or immune cells, through EC surface expression of adhesion molecules.^67^ An example of EC adhesion molecules is the family of selectins.^5^ Another example is intercellular adhesion molecule-1 (ICAM-1) which can interact with CTC or immune cell adhesion molecules, such as CD11a/CD18 (LFA-1) and others, to enhance CTC or immune cell migration through the endothelium.^95^ Lastly, through extracellular vesicles, ECs are able to send small lipid-enclosed particles to distant cells to effect physiological changes, locally or systemically. Extracellular vesicles are a relevant intercellular signaling mechanism that enables transfer of molecules between cells.^60^,^68^

All of these forms of intercellular interactions have been extensively reviewed in previous publications.^3^,^48^,^72^,^104^ Herein, for the purpose of exploring the role of the GCX in the mediation of intercellular interactions, we will focus on discussing Cx-containing gap junctions as a representative intercellular junction and the E-selectin endothelial surface receptor as a representative adhesion molecule.

### Intercellular Interactions Involving Gap Junctions

**Figure 3:**
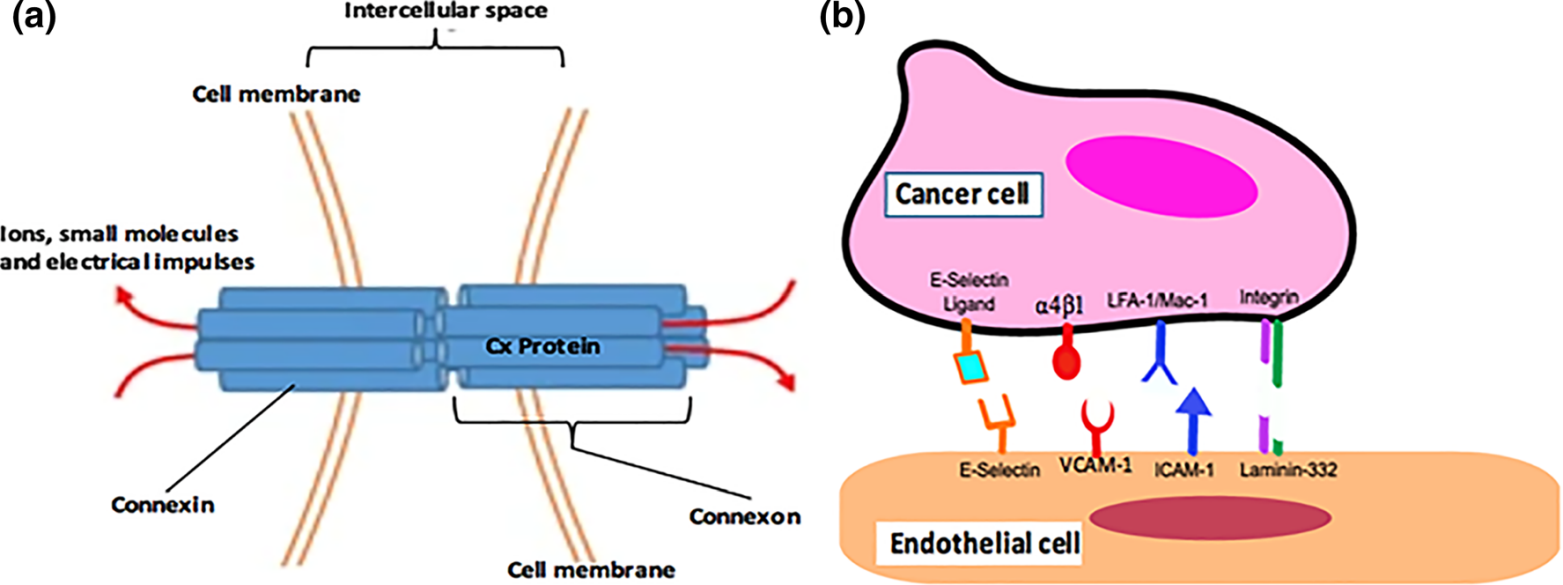
(a) Cx gap junction proteins form connexons. Two cells contribute one connexon each, which is docked through the cell membrane of the host cell. These connexons come together to form a gap junction protein able to transmit ions, small molecules and electrical impulses between two cells. *This is an adaptation of a previously published*^119^*figure that is used in accordance with the terms of the Creative Commons Attribution 4.0 International License (*http://creativecommons.org/licenses/by/4.0/*), which permits unrestricted use, distribution, and reproduction in any medium.* (**b)** Interaction between EC and cancer cell is mediated by receptor-ligand complexes. The ligands on the cancer cell align with the receptors on the surface of the EC for attachment. *This figure has been used with permission from the previous publisher.*^42^

Gap junctions pass through the cell membranes of adjacent cells, and they serve as a semi permeable pathway for the diffusion of ions and small molecules between the cells^119^ (Fig. 3a). These channels are made up of the previously mentioned transmembrane Cx proteins.^85^ Six Cxs from each adjacent cell membrane form a connexon (Fig. 3a). Two connexons contributed by adjacent cells come together to construct the cylindrical gap junction channel that becomes a mode of communication between the cells^8^ (Fig. 3a). Several types of Cx combinations may assemble to form the gap junctions between cells, and because of their short-life span they are renewed daily.^12^ In relation to ECs, three different Cx types are described: Connexin 37 (Cx37), Cx40 and Cx43.^23^,^41^,^121^ The relative amounts of expression of these Cx types depend on the vessel type.^26^,^46^ Relative expression of the different types of Cxs also depend on healthy versus diseased conditions, as described in a previously published review paper.^88^ In brief, healthy ECs mostly express Cx37 and Cx40. During the initiation of atherosclerotic lesions, Cx43 begins to be expressed in addition to Cx37 and Cx40.^12^ During the late stage of the disease only Cx43 is expressed indicating the drastic change in the Cx makeup of the vessel.^58^ In other diseases like cancer, there is growing evidence to suggest that expressed Cx regulates tumor growth.^1^,^66^ This regulation is known to happen at the transcription,^16^,^17^ post transcription^51^ and the protein synthesis levels.^110^ In cancer, the study of Cxs is very complicated because although ECs only express three Cxs, CTCs may express more than three Cx proteins (there are over twenty Cx proteins in the body). Regarding the functional roles of the Cxs, intercellular interactions mediated by Cxs are responsible for vasomotor responses and tone,^20^ with Cx40 being specifically known to be very important in vasoregulation to control blood pressure.^27^ The Cxs, especially Cx43, also play significant roles in intercellular adhesion, cell migration, and cell proliferation.^7^,^54^

### Intercellular Interactions Involving Adhesion Molecules

Interactions via adhesion molecules (Fig. 3b) are usually complicated and may involve multiple steps in a sequence to ensure intercellular binding. In general, three separate steps characterize the formation of adhesion molecule interactions, which are mediated by receptors and ligands. First, there is the primary recognition stage where receptors on one cell are recognized by corresponding ligands on another cell via electrostatic forces. Second, structural conformational changes and proper orientation occur to match ligands to their binding sites on receptors. Third, physical contact and binding are achieved, which creates a receptor-ligand complex between different cell types^40^,^81^ (Fig. 3b).

ECs have several surface receptors that initiate interactions with leukocytes, cancer cells, and other cell types (Table 1). These receptors include E-selectin, intercellular adhesion molecule-1 (ICAM-1), vascular cell adhesion molecule-1 (VCAM-1), integrins, and others^61^ (Fig. 3b and Table 1).

**Table 1:** EC receptors and their corresponding ligands on tumor cells.

EC receptor	Tumor ligand
E-Selectin	sLe^a^, sLe^x^, CD44^18^
VCAM-1	Alpha 4 Beta 1,7^84^,^101^
ICAM-1	LFA-1/Mac-, MUC-1^96^
Laminin-332	Integrin^109^
VEGFRs	Neuropilin-1^39^
PDGFRs	PGDF-B,C,D^31^
PECAM-1	TIMP-1^2^

During tumor invasion of the endothelium, these tumor ligands locate their respective receptors on the endothelium to initiate EC-Tumor interactions. These interactions result in firm attachment of tumor cells to ECs and also initiate biochemical changes with ECs and tumor cells. The activation of receptors or ligands have been used as markers for determining the aggressive nature of cancers. Abbreviations: sialyl Lewis^a^ (sLe^a^), sialyl Lewis^x^ (sLe^x^), cluster of differentiation 44 (CD44), lymphocyte function-associated antigen 1 (LFA-1), macrophage-1 antigen (Mac-1), mucin 1 (MUC-1), vascular cell adhesion molecule 1 (VCAM-1), intercellular adhesion molecule 1 (ICAM-1), vascular endothelial growth factor receptors (VEGFRs), platelet-derived growth factor receptors (PDGFRs), platelet endothelial cell adhesion molecule 1 (PECAM-1), platelet-derived growth factor (PGDF), tissue Inhibitor of metalloproteinase 1 (TIMP-1)

Of these adhesion receptors, E-selectin is of particular interest because it is the first EC adhesion molecule to interact with CTCs during cancer or leukocyte invasion in atherosclerosis. E-selectin belongs to a group of selectins which are glycoproteins that mediate circulating blood cell attachment to the endothelium^56^ (Fig. 3b). E-selectin is mostly expressed by ECs via endothelial activation during inflammation or in the presence of cytokines.^56^ As a consequence, E-selectin can promote atherosclerosis and cancer metastasis progression leading to poor prognosis of disease.^49^,^55^ Gakhar *et al* reported that in cancer, CTCs isolated from men with castration-resistant prostate cancer exhibited significant physical (tethering and firm adhesion) interactions with E-selectin-coated surfaces.^34^ These interactions were diminished when E-selectin antibodies were present.^34^

## The Endothelial Glycocalyx: Its Structure

**Figure 4:**
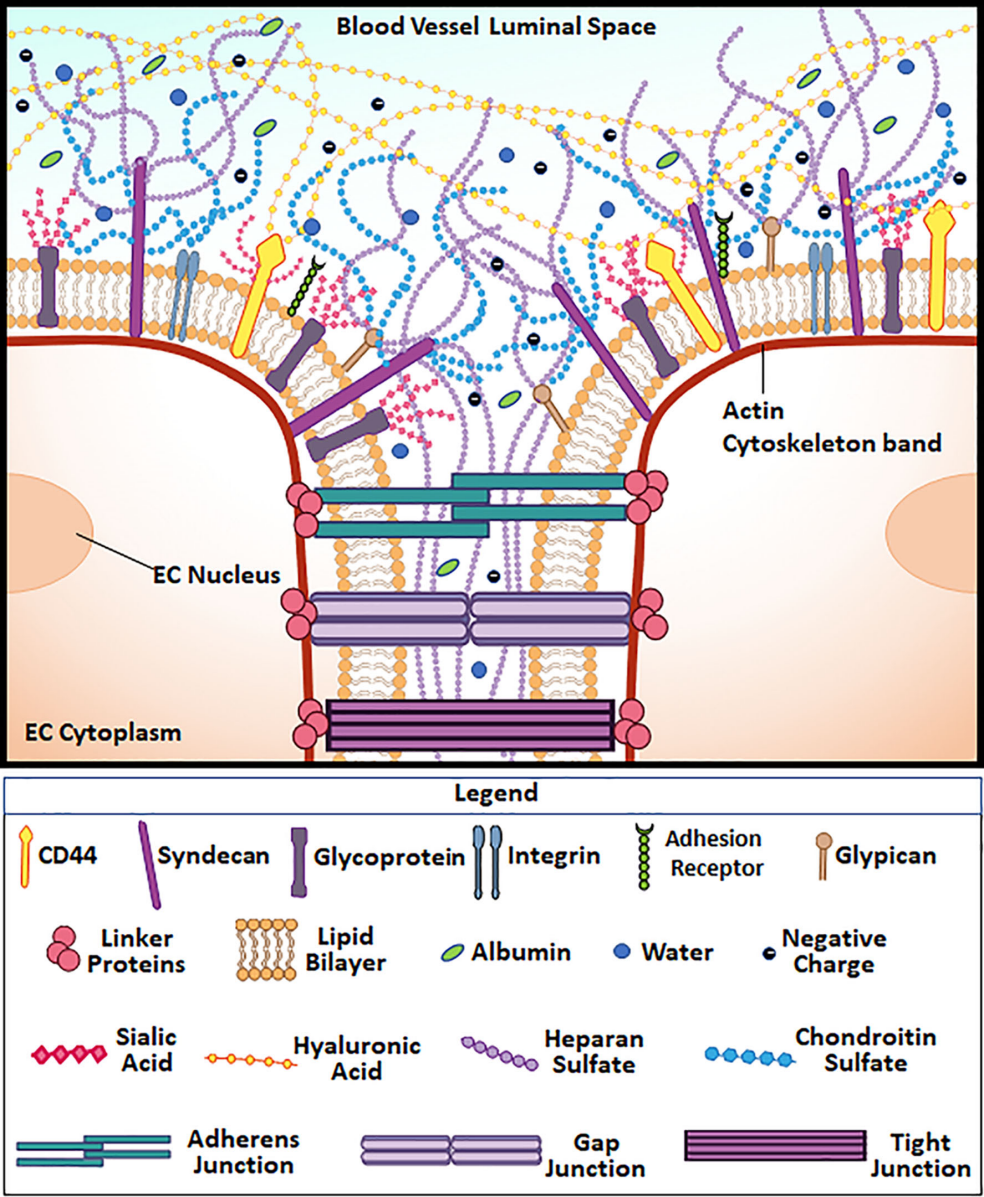
Physical structure and components of the EC GCX. Image depicts the various GCX components like HS, SA *etc*. The GCX extends from the endothelial cell membrane to the lumen of blood vessel interacting with circulating cells in the blood. The image also depicts the GCX extending between adjacent cells and interacting with the junctional proteins like gap junctions and tight junctions. The image also shows the height disparity between adhesion receptors like E-selectin and integrin and the GCX.

The gap junctions and adhesion molecules both interface with and are embedded in the GCX on the EC surface (Fig. 4). The GCX is a sugar-rich layer that has been seen to encapsulate ECs (Ref. 30; Harding et el, unpublished data), although most visualization methods detect GCX predominantly on the luminal side of ECs. It is connected to EC membrane through several backbone molecules,^83^ mainly proteoglycans and glycoproteins like syndecans and glypicans, as indicated in Fig. 4. These backbone molecules have sugar chains covalently or loosely linked to them. The glycosaminoglycan (GAG) sugar chains, characterized by distinct disaccharide unit repeats, include hyaluronic acid (HA), heparan sulphate (HS), and chondroitin sulphate (CS). HA are long GAG chains attached to EC membrane bound receptors, such as CD44 (Fig. 4), and are presumed to intertwine through GCX and provide a scaffold for the GCX.^24^ The HS GAG is the dominant constituent of GCX (Fig. 4). HS is a linear sulfated polysaccharide chain and anchored to the syndecan and glypican core proteins.^37^ The CS GAG is also an abundant GAG and is bound to syndecan alongside HS. CS is covalently linked to its core protein via the GAG-protein linkage.^57^ The ratio of HS to CS is reported to be in the order of 4:1. The combination of HS and CS plays a very critical role in the structural stability of GCX.^77^,^122^ In addition to the GAGs, a sialoglycoprotein, sialic acid (SA) (Fig. 4), also commonly associates with the EC GCX. SA consists of complex sugar units and is mostly located at the innermost part of GAGs (Fig. 4). Given its location in proximity to the EC surface adhesion molecules, SA should play a significant role in maintaining the barrier integrity of GCX.^21^,^112^ SA has the added advantage of being negatively charged and, therefore, engages in the repulsion of unwanted intercellular and molecular interactions from components of the blood circulation.^112^ The arrangement of the GCX components determines overall GCX form and function.^106^

## Endothelial Glycocalyx: Its Functions and Implications for Gap Junction and Adhesion Molecule Mediated Intercellular Interactions

Several reports have highlighted the following functions of the GCX: mechanotransduction,^123^ barrier protection^124^ and regulation of permeability, and other functions. Regarding mechanotransduction, due to the transmembrane nature of GCX core proteins, Tarbell and colleagues have noted that the GCX transduces shear and stretch forces into biomolecular responses of ECs.^105^ The role of GCX in mechanotransduction was initially suspected when it was discovered that GCX is anchored to a scaffold of cytoskeleton actin filaments that form an actin cortical web.^99^ The barrier and permeability properties of GCX are usually deduced from the rate at which small molecules like dextran and other tracers as well as leukocytes and CTCs move into the GCX and across the endothelial layer.^24^

Since the cytoskeleton actin web is in close proximity to and, in some cases, linked to transmembrane adhesion molecules and gap junction proteins, it has been presumed that GCX is an important factor in transducing mechanical forces into changes in intercellular interactions. Thi and colleagues previously studied the transmission of fluid shear stress through the GCX to the actin cortical web of the cytoskeleton.^107^ They discovered that, with intact GCX, fluid shear stress induces F-actin distribution primarily to the cell borders where junctional proteins like Cx are located.^107^ Using a GCX digesting enzyme that specifically targets the HS GAG of GCX, it was shown that the absence of HS GAG results in disorganization of the actin filaments and loss of Cx proteins under shear stress.^107^ Conversely, reinforcing the HS component of GCX by adding fetal bovine serum and albumin to the culture medium in addition to prescribed shear stresses resulted in dramatic enhancement of the actin cortical web and expressed Cx.^35^,^107^ Due to the observed GCX-actin-Cx protein relationship, it was proposed that functional performance of Cx-containing gap junction channels would also be enhanced and lead to active cell-to-cell communication between ECs in flow conditions. We recently proved this to be true.^69^

In addition to GCX’s suspected role in regulating gap junctions, we speculate that the GCX is relevant in regulating adhesion molecules on the surface of the endothelium thereby regulating blood circulating cell accessibility to the endothelial surface. Interaction between ECs and white blood cells, for example, is adhesion molecule mediated and governed by the availability of receptors, such as E-selectin, which bind to the ligands on white blood cells. Weinbaum and coworkers previously reported that the ability of immune cells and other cells to penetrate the GCX layer to access the adhesion receptors is dependent on the porosity and stiffness of the GCX.^116^ GCX thickness relative to the length of the receptors on the endothelial surface is also important in determining if receptors are shielded from ligands on circulating cells.^24^,^30^,^98^ Enzymatic degradation of GCX could be a possible mechanism through which EC receptors are exposed to circulating cell ligands for the formation of intercellular connections. In support of this idea, it has been shown that by suppressing the activities of matrix metalloproteases, a class of enzymes reported to degrade GCX, leukocyte-EC interactions can be inhibited.^76^ Recent work has shown that in addition to enzymatic degradation of GCX, hemodynamic factors could also result in the degradation of GCX. In areas of the vasculature where disturbed flows exist, the GCX is degraded significantly compared to uniform flow (UF) areas of the vasculature.^43^ Therefore, flow-induced degradation of the GCX could be another mechanism through which EC surface adhesion molecules are exposed. We recently showed that both enzymatic and flow induced degradation of the GCX, which exposes the adhesion molecules, significantly increases EC adhesiveness to circulating cells.^70^,^71^

## Emerging Data on the Implications of Glycocalyx-Mediated Intercellular Interactions for Health, Atherosclerosis, and Cancer

Relevant to understanding the underlying cellular and molecular causes of atherosclerosis and cancer, to date, not much effort has been made beyond what we describe above to explain the role of the GCX in intercellular interactions. Therefore, the evidence provided above draws an incomplete picture of the connection between gap junction functionality and GCX health, it provides limited evidence that GCX regulates adhesion molecule interactions between ECs and blood circulating cells. Therefore, our lab has sought to more firmly elucidate the role played by GCX in intercellular interactions, specifically related to how GCX mediates EC-to-EC communication and EC-to-CTC attachment in atherosclerosis and cancer, respectively. We have been testing the hypothesis that GCX dysfunction results in impairment of gap junction activity while also promoting adhesion molecule accessibility, for disrupted interendothelial communication and increase in CTC attachment, clustering and migration through the endothelium. Here, we summarize our investigations that were performed to test this hypothesis.

**Figure 5:**
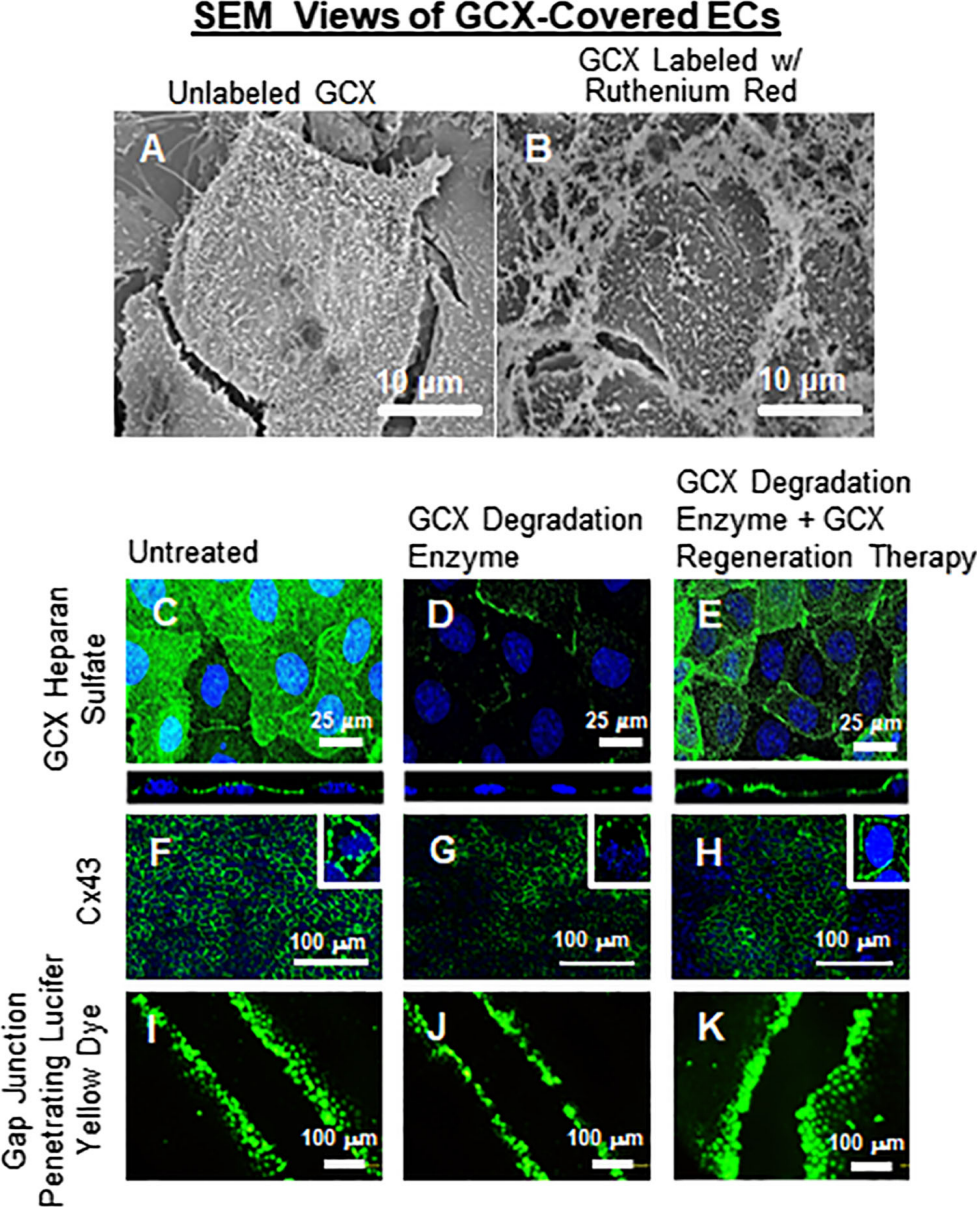
Summary of the impact of GCX degradation on inter-endothelial communication. (**a)** SEM image showing Unlabeled single RFPEC. (**b)** SEM image of RFPEC labeled with ruthenium red, image depicts extracellular structures located mostly at the cellular junctions. These structures are suspected to be GCX. (**c)** Untreated (control) RFPEC show intact HS at baseline conditions (green is HS with blue 4’6-diamidino-2-phenylindole staining the cell nucleus). (**d)** With 25 μIU/ml of Hep III, HS is degraded. (**e)** Combined treatment of exogenous HS and S1P affected HS expression HS by restoring baseline conditions. (**f)** Cx43 expression in control RFPEC. (**g**) With 25 μIU/ml of Hep III, Cx43 is significantly degraded in RFPEC. (**h)** Combined treatment of exogenous HS and S1P results in significant restoration of the expression of Cx43 in RFPEC. (**i)** Control Lucifer Yellow Dye Transfer between RFPEC. (**j**) 25 μIU/ml of Hep III results in the blockage of Lucifer Yellow Dye across RFPEC. (**k)** Combined treatment of exogenous HS and S1P results in the restoration of the Cx43 which enhances the transfer of Lucifer Yellow Dye among neighboring RFPECs.

We recently investigated the role of GCX and its HS component in regulating the expression of Cx43-containing gap junctions at EC borders as characterized immunocytochemically, and in regulating the function of Cx-containing gap junctions as assessed by measuring interendothelial spread of gap junction permeable Lucifer Yellow dye (Fig. 5).^69^ These studies were performed using cultured rat fat pad ECs (RFPECs) expressing an intact GCX or a GCX with enzymatically degraded HS, a major component of GCX. For some EC cultures, a novel GCX recovery approach was employed in an attempt to regenerate lost HS and to further investigate the importance of HS for Cx43 functionality. The ECs were treated with exogenous HS with or without the GCX regenerator and protector sphingosine 1- phosphate (S1P). The results of this study demonstrated that, with intact GCX (Fig. 5c), 60% of EC borders expressed immunocytochemically labeled Cx43 (Fig. 5f) and Lucifer Yellow dye spread to 2.88 ± 0.09 neighboring cells (Fig. 5i). HS degradation (Fig. 5d) decreased Cx43 expression to 30% (Fig. 5g) and reduced dye spread to 1.87± 0.06 cells (Fig. 5j). Artificial HS recovery with exogenous HS partially restored Cx43 expression to 46% and yielded dye spread to only 1.03 ± 0.07 cells. Treatment with both HS and S1P, recovered HS and the GCX (Fig. 5e), and restored Cx43 to 56% (Fig. 5h) with significant dye transfer to 3.96 ± 0.23 cells (Fig. 5k). This study, reported in a recent peer-review paper^69^ and published in a patent application (US 2020/0023001 A1), shed light on the role of GCX in enabling EC-to-EC communication function which is lost during the progression of certain cardiovascular related pathologies including cancer and atherosclerosis.

We also investigated the importance of GCX in concealing or uncovering receptors that mediate cancer-endothelial cell interactions.^71^ While it is known that cancer cell interactions with vascular ECs drive metastatic cancer cell extravasation from blood vessels into secondary tumor sites, the mechanisms of action are still poorly understood. This investigation added fundamental information to the body of knowledge about the mechanisms underlying cancer-EC interactions. Specifically, we tested the hypothesis that neuraminidase-induced degradation of EC GCX, particularly the sialic acid (SA) residue components of the GCX, substantially increases metastatic cancer cell attachment to ECs. Our interest in the SA component of the EC GCX was based on the fact that SA in the cancer cell GCX is a marker of oncogenesis and tumor survival and on the fact that the SA-degrading enzyme, neuraminidase, is strongly upregulated with cancer metastasis and other pathologies, yet the role of SA in the endothelial GCX during oncogenesis has been understudied.^78^,^82^,^113^,^115^ We investigated the effect of dose dependent administration of SA-degrading neuraminidase on RFPECs. After administering the enzyme, immunostaining and confocal microscopy were used to investigate structural and morphological changes in the overall GCX versus the α‐2,6‐linked and α‐2,3‐linked SA residues of the GCX. In addition, we investigated the effect of the presence of the enzyme on the attachment of 4T1 breast cancer cells to the endothelium. To our knowledge, our study is the first to isolate the role of GCX SA residues in cancer cell attachment to the endothelium, which were found to be differentially affected by the presence of neuraminidase, in correlation to variations in metastatic cancer cell homing to ECs. Reported in a recent paper,^71^ this study provides an improved understanding of the effect of GCX degradation on the attachment of cancer cells to the endothelium, which is very much needed for creating therapeutic measures that will combat the spread of cancer via strengthening of GCX against GAG degrading cytokines released by cancer tumors.

**Figure 6:**
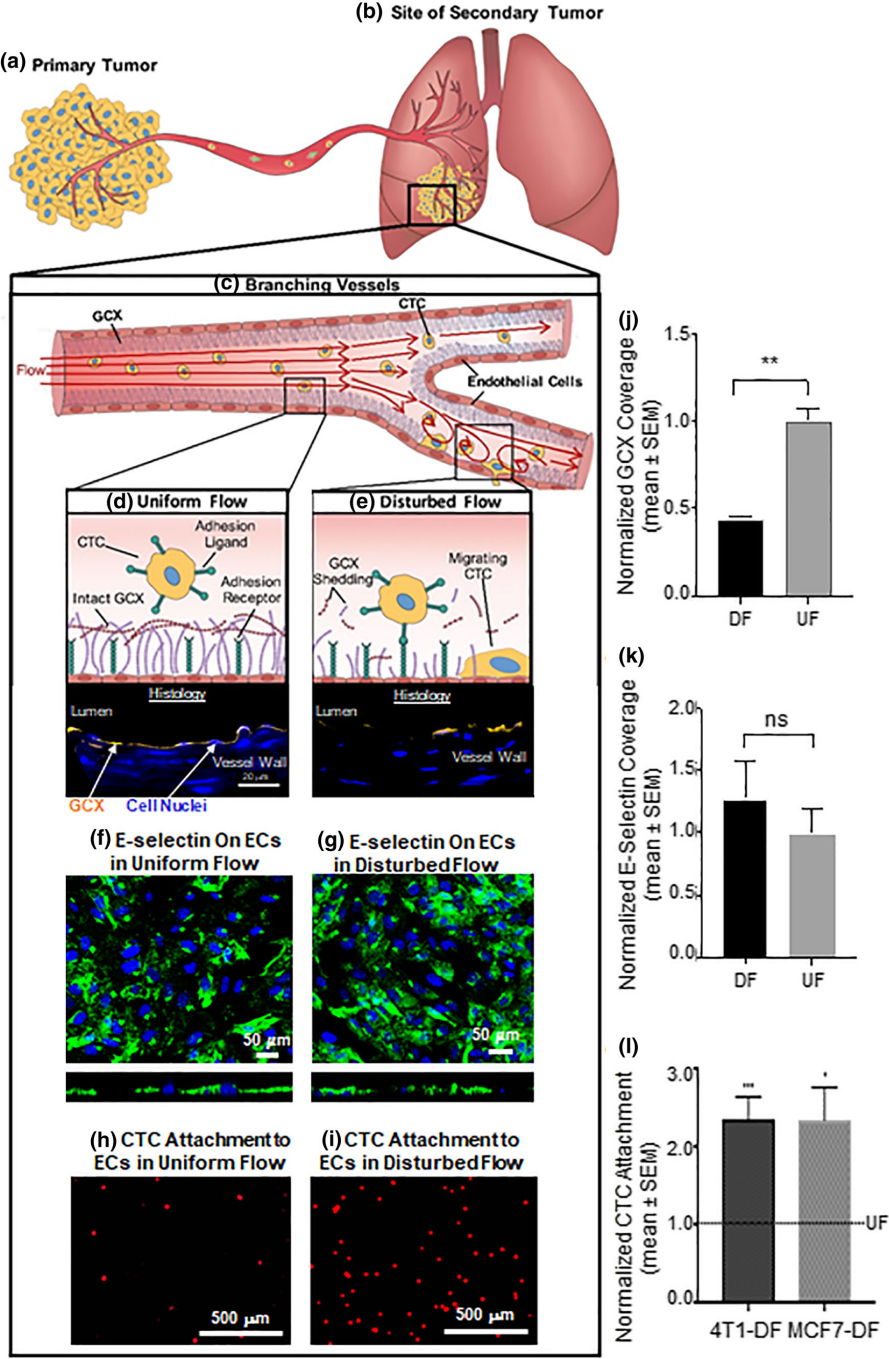
Summary of the impact of flow dependent GCX degradation on endothelial-CTC interactions. (**a)** CTCs (yellow cells with blue nuclei) leave the primary tumor, and (**b)** form secondary tumors via blood vessels, by penetrating the EC barrier (red cells with dark red nuclei). (**c)** Geometric changes within blood vessels result in different flow patterns. (**d)** Blood vessel walls exposed to uniform flow (UF) have intact GCX, preventing CTC attachment to the endothelium. (**e)** Vessel branching produces flow disturbances (DF) that can degrade the endothelial GCX, and we hypothesize that this makes ECs accessible to CTCs. (**f)** UF-conditioned HUVEC stained for E-selectin. (**g)** E-selectin expression in DF-conditioned HUVEC. (**h)** MCF-7 breast cancer cell (red) attachment to UF-conditioned HUVEC. (**i)** Increase in MCF-7 cell (red) attachment to HUVEC after exposing HUVEC to DF. (**j)** Quantification for GCX expression in DF versus UF conditioned vessels. (**k)** Quantification of E-selectin expression on HUVEC, depicting a non-significant difference in expression of E-selectin on DF versus UF-conditioned HUVEC. (**l)** Quantification of attached mouse breast cancer cells (4T1) and human breast cancer cells (MCF-7) in DF conditions, in comparison to UF conditions.

As it is known that GCX structure depends on vascular flow patterns, which are irregular in tumor environments, we performed another study to obtain evidence that disturbed flow (DF) induces GCX degradation and leads to CTC homing to the endothelium (Fig. 6).^70^ We used a customized flow chamber to introduce disturbed and uniform flow (DF and UF) patterns to ECs, mimicking dynamic *in vivo* flow conditions. The specific flow parameters that were generated by our flow chamber, for DF conditions, included a substantial spatial shear stress gradient such that magnitude of shear stress ranged from -8 to 12 dynes/cm^2^. Other DF parameters included flow reversal, flow stagnation at the center of bi-directional flow, and flow adaptation to steady conditions. The UF parameters simply consisted of unidirectional flow with zero shear stress gradient and a constant 12 dynes/cm^2^ magnitude of shear stress. DF and UF were both in the laminar flow regime. The effect of DF vs. UF on the changes in GCX structure and morphology was assessed with immunostaining and confocal microscopy, using human umbilical vein ECs (HUVECs). This was followed by EC-CTC attachment experiments, using breast cancer cells from mice (4T1) or human (MCF7) donors, to determine the effect of different flow conditions on the early steps in secondary tumor formation: attachment to the endothelium, clustering, and migration of cancer cells across the endothelium. Our *in vitro* results demonstrated that a 2-fold greater attachment of CTCs to human ECs occurred in DF conditions, compared to UF conditions (Figs. 6h, 6i, and 6l). These results corresponded to an approximately 50% decrease in wheat germ agglutinin (WGA) labeled components of the GCX in DF conditions, versus UF conditions (Figs. 6d, 6e, and 6j; WGA labels the SA component of the GCX, primarily, but also has an affinity for other GCX components^86^,^92^,^102^). E-selectin receptor expression was similar in DF and UF conditions (Figs. 6f, 6g, and 6K). These results suggest that the low level of CTC-EC interactions in UF can be attributed to the abundance of the protective GCX. We confirmed the role of the GCX both *in vitro* and *in vivo.* Neuraminidase enzyme was applied to degrade WGA-labeled GCX in UF cell culture conditions and in Balb/C mice. This led to an over 2-fold increase in CTC attachment to cultured ECs and to Balb/C mouse lungs, respectively, compared to non-enzymatic conditions. This study is described at length in a previous publication,^70^ and has increased our understanding of the role played by vascular geometry and flow parameters on GCX structure and morphology as well as the effect of such flow patterns on GCX-mediated EC-cancer cell interactions.

## Summary of Work and Future Perspectives

Although the role of GCX in regulating intercellular interactions has been understudied, the recent work published by our group proves that there is a correlation between the structural integrity of the GCX and the performance of intercellular interaction proteins like Cx43 and E-selectin.^69^–^71^,^107^

**Figure 7:**
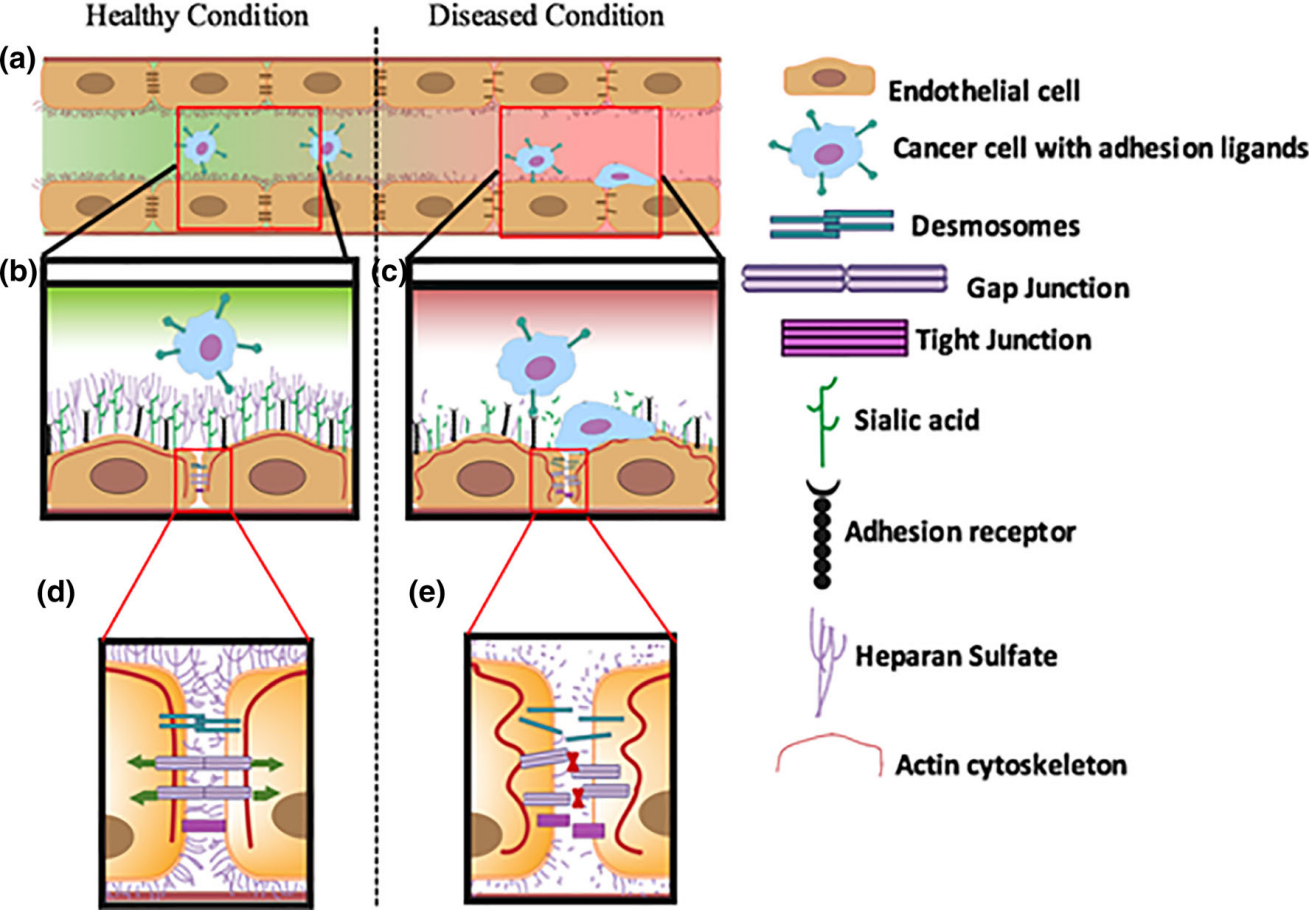
Conceptual depiction of the role played by endothelial glycocalyx in intercellular interactions. (**a)** Vessel showing healthy and diseased conditions. On the left side the vessel is healthy and characterized by intact GCX which ensures proper cell-to-cell communication between adjacent endothelial cells (ECs). The healthy GCX also prevents CTCs from attaching to adhesion receptors on the surface of endothelial cells. The right side shows a diseased condition where GCX is degraded leading to lack of cell-to-cell communication and attachment of CTCs to the endothelium. (**b)** Zoom-in of healthy endothelial GCX and stable actin cortical web. The GCX prevents ligands on CTCs from binding to the receptors on the endothelial surface. The actin cortical web is stable, ensuring proper alignment of junctional proteins. (**c)** Zoom-in of diseased GCX and destabilized actin cortical web. The degraded or diseased GCX uncovers the adhesion receptors on the surface of the endothelium for easy binding to ligands on CTCs. Destabilized actin cortical web leads to misaligned junctional proteins. (**d)** Zoom-in of healthy junctional proteins, showing active communication between adjacent ECs. (**e)** Degraded GCX leading to a destabilized actin cortical web, which disrupts junctional protein alignment and prevents intercellular communication between adjacent ECs.

We have shown that the opening and closing of Cx containing gap junctions, especially Cx43 containing gap junctions, are directly dependent on the health of GCX.^69^ In healthy conditions (Figs. 7a, 7b, and 7d) the GCX is stable and Cx43 is properly aligned to its adjacent Cxs, while connexons are also aligned to adjacent connexons, enabling the transport of ions and molecules through gap junctions that connect adjoining cells. However, in diseased conditions, degraded GCX destabilizes Cx43, the connexons and gap junctions, and prevents the transport of ions and molecules (Figs. 7a, 7c, and 7e).

Further studies are necessary to investigate the role of GCX in gap junction mediated intercellular communication. The formation of Cx-containing gap junctions includes oligomerization, trafficking, the actual gap junction formation, gating function and internalization.^25^,^52^,^97^ Of these sequences, we have only shown the connection between GCX and the gating function of Cxs. Due to reports by Mia Thi *et al* and others^93^,^108^ in the involvement of cytoskeleton in Cx expression, could it be possible that the GCX can be implicated in the trafficking and internalization of Cx-containing gap junctions? This remains to be clarified. In addition, the formation of gap junctions could be occurring as a result of the heteromeric combination of different types of Cx proteins.^22^,^45^,^50^,^100^,^111^ While we have only studied GCX regulation of one type, Cx43. It will be very interesting to compare the role of GCX in regulation of homomeric (single or similar subunit of Cx protein) versus heteromeric (different subunits) Cx containing gap junctions. Such an experiment would broaden our understanding of the role played by GCX in modulating cell-to-cell communication.

It has been proposed, and we have shown, that accessibility of E-selectin receptors on the surface of the endothelium for easy binding of ligands on circulating cells is GCX dependent (Figs. 7a and 7b).^70^,^71^ In disease conditions, degraded GCX enhances the interactions between ECs and CTCs in a manner that may result in secondary tumor initiation (Figs. 7a and 7c). We further showed that the SA component of the GCX plays a significant role in the process of concealing these receptors from CTCs.^71^ It is necessary to study GCX involvement in the attachment of circulating cells at specific stages of the process: slow rolling, adhesion, firm binding, crawling and paracellular and transcellular migration.^62^,^63^ Each of these stages is mediated by a different form of receptor on the surface of the endothelium (Table 1). E-selectins, which we have studied, are only reported to be important in slow rolling of circulating cells on the endothelium.^91^ Future work should investigate the full class of selectins, which in addition to E-selectin include P-selectin and L-selectin. These selectins could be differentially regulated by GCX. Another adhesion molecule, integrin, is worth investigating because of the specific role played by integrins in creating firm adhesion complexes during immune and cancer cells interactions with the endothelium.

Lastly, GCX is composed of different GAGs and numerous other components, as previously mentioned. We have shown the importance of SA and HS in regulating intercellular interactions in regard to cell-to-cell communication and cell-to-cell adhesion. Perhaps other components that form an integral part of GCX should be studied to understand their role in intercellular interactions. Investigating the full range of GCX components is particularly important for meeting a two-tiered goal: (1) Better understand the GCX role in intercellular interactions and, (2) Develop GCX strengthening drugs to prevent unwanted GCX degradation in a component-specific manner, to stop disease progression. Achieving this goal will significantly advance the field.

## Abbreviations

Cx: Connexin
CS: Chondroitin sulfate
CTC: Circulating cancer cells
DF: Disturbed flow
ECs: Endothelial cells
eNOS: Endothelial nitric oxide synthase
GCX: Endothelial glycocalyx
GAGs: Glycosaminoglycans
HA: Hyaluronic acid
Hep III: Heparinase III
HS: Heparan sulfate
HUVEC: Human umbilical vein endothelial cell
ICAM-1: Intercellular adhesion molecule 1
MCF7: Human breast cancer cells
PECAM-1: Platelet endothelial cell adhesion molecule-1
RFPEC: Rat fat pad endothelial cell
SA: Sialic acid
S1P: Sphingosine 1-phosphate
UF: Uniform flow
VCAM-1: Vascular cell adhesion molecule 1
WGA: Wheat germ agglutinin lectin
4T1: Stage IV Metastatic Breast Cancer Cells
